# Identifying novel glioma associated pathways based on systems biology level meta-analysis

**DOI:** 10.1186/1752-0509-7-S2-S9

**Published:** 2013-12-17

**Authors:** Yangfan Hu, Jinquan Li, Wenying Yan, Jiajia Chen, Yin Li, Guang Hu, Bairong Shen

**Affiliations:** 1 Center for Systems Biology, Soochow University, Suzhou 215006, China; 2 The First Affiliated Hospital of Soochow University, Suzhou 215006, China; 3 School of Chemistry and Biological Engineering, Suzhou University of Science and Technology, 215009, China; 4 Department of Bioinformatics, School of Medicine, Soochow University, Suzhou 215123, China

## Abstract

**Background:**

With recent advances in microarray technology, including genomics, proteomics, and metabolomics, it brings a great challenge for integrating this "-omics" data to analysis complex disease. Glioma is an extremely aggressive and lethal form of brain tumor, and thus the study of the molecule mechanism underlying glioma remains very important. To date, most studies focus on detecting the differentially expressed genes in glioma. However, the meta-analysis for pathway analysis based on multiple microarray datasets has not been systematically pursued.

**Results:**

In this study, we therefore developed a systems biology based approach by integrating three types of omics data to identify common pathways in glioma. Firstly, the meta-analysis has been performed to study the overlapping of signatures at different levels based on the microarray gene expression data of glioma. Among these gene expression datasets, 12 pathways were found in GeneGO database that shared by four stages. Then, microRNA expression profiles and ChIP-seq data were integrated for the further pathway enrichment analysis. As a result, we suggest 5 of these pathways could be served as putative pathways in glioma. Among them, the pathway of TGF-beta-dependent induction of EMT via SMAD is of particular importance.

**Conclusions:**

Our results demonstrate that the meta-analysis based on systems biology level provide a more useful approach to study the molecule mechanism of complex disease. The integration of different types of omics data, including gene expression microarrays, microRNA and ChIP-seq data, suggest some common pathways correlated with glioma. These findings will offer useful potential candidates for targeted therapeutic intervention of glioma.

## Background

In the last few years, the post-human genome project era is coming, which has witnessed the evolution of multi-level omics data, including genomics, proteomics, and metabolomics. As more and more microarray datasets and technologies development, they have gradually become standard resources and tools to analysis complex disease. On the other hand, cancer is a complex biological system and thus its molecular mechanism needs to be understood at systems-level [1,2]. As a most recent development, micro-RNA (miRNA) not only has promising clinical applications in cancer diagnosis and treatment, but also could as competing endogenous RNAs (ceRNA) to construct a regulation network to understand regulatory pathways in cancer [3]. Therefore, the meta-analysis [4] of cancer by integrating omics data at the systems biology level is of significant importance, or at least, is possible.

Brain tumours are kind of complex cancer and high leading cause of death in the United States. Glioma, the most common type of primary brain tumours, which occurs in the glical cells of adults [5,6]. According to their histological types and World Health Organization (WHO) grades [7], gliomas can be classified into several general categories, for example glioblastomas multiforme (GBM) belongs to a WHO grade IV tumor. Till now, most of research effort has been directed at identification of important genes in glioma. In 2010, Katara et al. [8] suggested that CDK4, MDM2, EGFR, PDGFA, PDGFB and PDGFRA genes can be served as biomarkers for glioma. In addition, they also found that CDKN2A, PTEN, RB1 and TP53 are the tumor suppressor genes. Li et al. [9] found that ECRG4 is a down-regulated gene in glioma, which has been reported as a candidate tumor suppressor in other cancers. However, the study of molecular bias of glioma at the system level is still needed [10].

In order to improve therapeutics of glioma, it will require greater knowledge at both the genomic and transcriptional level. Fortunately, recent advances show that miRNA expression profiles provide valuable molecular signatures for gliomas. Han et al. [11] reported that miR-21 could enhance the chemotherapeutic effect of taxol on human glioblastoma (GBM) U251 cells. Chromatin immunoprecipitation followed by high-throughput sequencing (ChIP-seq) technology has also been applied to analysis GBM cells, such as identify global SOX2 binding regions [12]. Token these data together, it is possible to analyse the glioma at the systems biology level, from pathway level, network level, and even to system network dynamics level.

In this paper, we aimed to analyze the molecular basis of glioma at systems biology level, by integrating three types of omics data, including gene expression microarray, MicroRNA and ChIP-seq data sets. The novel statistical method, named Cancer Outlier Profile Analysis (COPA) [13], was used to detect the significantly differentially expressed genes. Furthermore, the pathway enrichment analysis, Gene Set Enrichment Analysis (GSEA) [14], and MAPE [4] approach were also performed, and some possible pathways that may be related to disease are found in glioma.

## Results

### Data collection

We have downloaded the raw gene expression data sets on glioma from Gene Expression Omnius (GEO), a public database at NCBI. The detailed information of these four datasets is summarized in Table 1. According to WHO standard, the gliomas were pathologically diagnosed to subtypes, which include 42 normal brain samples and 462 patient tumor samples.

**Table 1:** Information on microarray expression profiling data of glioma

Dataset	Platform	Sample Number	Sample Information						Gene Number
				
			Normal	Tumor					
					
				Astrocytic		Glioblastomas	Oligodendrogliomas		
							
				PA	A		OD	OA	
Data 1	HG-U95Av2	25	5	6	/	7	7	/	12625

Data 2	U133-Plus 2.0 Array	284	8	8	29	159	52	28	54675

Data 3	U133-Plus 2.0 Array	15	6	/	/	8	1	/	54675

Data 4	U133-Plus 2.0 Array	180	23	/	26	81	50	/	54675

### Microarray statistical analysis for glioma datasets

It is well known that tumor heterogeneity is a generic property for cancer including glioma, which will reflect its evolutionary dynamics [15]. Traditional statistics, such as t-statistic and SAM [16,17], will not work for detecting multiple coexisting genes caused by the heterogeneity of cancer. In order to address this problem, a novel but powerful method called COPA was used here to meta-analyze the expressed gene datasets. Meta-analysis is a statistical technique to combine results from several microarray studies, increasing the reliability and robustness of results from individual studies. COPA is proposed by MacDonald et al. [13] by adding a simple test based on robust centering and scaling of the data to standard statistical tests.

First of all, the samples were classified into two types: Normal and Glioma, for the detection analysis in the framework of COPA. The glioma sample can be further classified into several subgroups, and 12 groups in all were selected for the COPA analysis. The numbers of significant genes of all datasets were close at the value of 1.8, which was set as the COPA threshold to define the 'outlier' status in the cancer samples. The text-mining searches in the Entrez PubMed database found that 853 out of 6306 (14%) genes were related to glioma.

**Figure 1:**
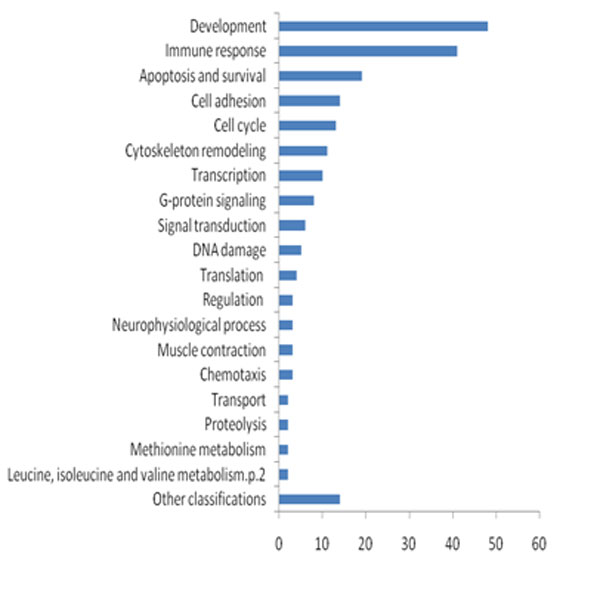
**GeneGO Ontology classification of 213 enriched pathways**.

Then the pathway enrichment analysis was performed by mapping these differentially expressed genes to GeneGO's MetaCore™, a manually curated and comprehensive commercial database. MetaCore™ is the flapship product of GeneGo, which acts an integrated software suite for functional analysis of experimental data, such as human protein-protein, protein-DNA and protein compound interactions, metabolic and signalling pathways for human, mouse and rat. Accordingly, a total of 213 pathways were emerged in GeneGO database, which have p value less than 0.05. Figure 1 shows the GeneGO's Ontology categories of these 213 pathways. In particular, 48 pathways were related to developmental process, 41 pathways were relevant to immune response, as well as 19 pathways of them were associated with apoptosis and survival.

In addition, pathway analysis (or gene set analysis) was method for correlating the known microarray genes with the defined genes from biological pathway databases. The Gene Set Enrichment Analysis (GSEA) is an improved pathway analysis, which was performed to judge which gene set/pathway is significant among the datasets [14]. Herein, the C2 curated gene sets from the Molecular Signature Database (MSigDB) was selected as the gene set annotations, and then we got 513 outlier gene sets with p value less than 0.05.

### Signature similarities at the system level are higher than that at the gene level

**Figure 2:**
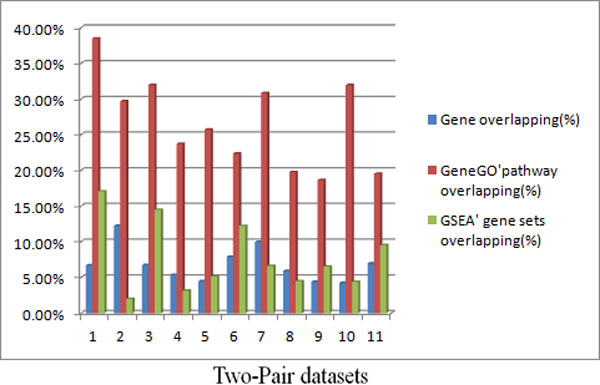
**Comparisons of overlapping analysis**. Bar plot of the percentage overlapping on gene and pathway/gene sets levels across 11 pairwise datasets. The blue bars stand for the percentage overlapping of differentially expressed genes, while red bars and green bars stand for percentage overlapping of enriched pathways in GeneGO database and enriched gene sets in GSEA, respectively.

As our pervious works proposed [18], the similarity of signature at the pathway/gene set level is higher than that at the gene level. In the same way, the overlapping analysis both at the gene level and pathway/gene set level was implemented. For the four datasets, 11 pairs of datasets could be comparable, according to the different stages of the glioma. Comparisons of the overlapping percentage among differentially expressed genes, pathways enriched by GeneGO's database, and gene sets enriched by GSEA are shown in Figure 2. The result clearly showed that the consistency across studies was higher at the pathway/gene set level than at the gene level. The p-value for the difference of overlapping between outlier genes and GeneGO's enriched pathways was 2.77e-07 by paired t-test. The overlapping of gene sets evaluated by GSEA software indicated that 64% of the pairwise data sets are more overlapped at the gene set level than that at the gene level. Therefore, these two analyses both verified our pervious hypotheses that mentioned in the beginning of this section.

### Identification of novel pathways by pathway level meta-analysis

**Figure 3:**
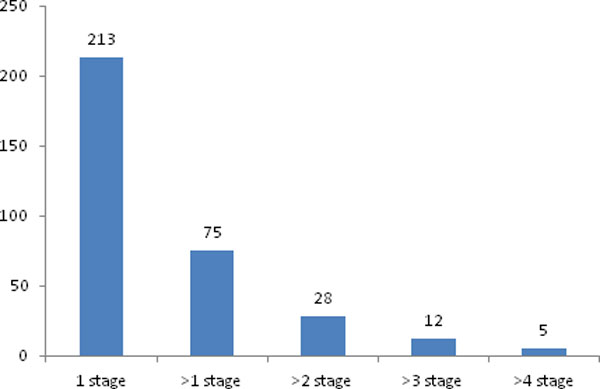
**Number of enriched pathways overlapped by various stages calculated based on GeneGO analysis**. The column lines described the number of enriched pathways that were overlapped by one stage, at least one stage, at least two stages, at least three stages, and at least four stages, respectively.

From the above result, we knew that the overlapping of the enriched pathways was much higher than that for the individual gene. In comparison with the gene level, the identified pathways at pathway level were predominantly more robust and closer to the phenotype of interest. The number of enriched pathways obtained from GeneGo in the four datasets classified by grades has been compared, as shown in Figure 3. We found that 12 common pathways are shared by at least four stages, as listed in Table 2. When checking the results in PubMed, the top 6 pathways have been confirmed to be associated with glioma.

**Table 2:** The 12 GeneGO's pathways shared by at least four stages among datasets

Pathway Name	Pubmed Count*
Chemokines and adhesion	687

Cell cycle (generic schema)	1122

TGF, WNT and cytoskeletal remodeling	380

WNT signalling pathway. Part 1. Degradation of beta-catenin in the absence WNT signalling	44

WNT signalling pathway. Part 2	44

Cytoskeleton remodeling	1

Role of IAP-proteins in apoptosis	0

Regulation of G1/S transition (part 1)	0

NOTCH1-mediated pathway for NF-KB activity modulation	0

Regulation of epithelial-to-mesenchymal transition (EMT)	0

TGF-beta-dependent induction of EMT via SMADs	0

Non-genomic (rapid) action of Androgen Receptor	0

Table 3 demonstrates the other six pathways that have not been reported as glioma related pathways. For these pathways, we further investigated the number of identified genes and all genes. As expected, some indirect evidences ware also found to support our results. From the Table 3, one can see that a large number of reported expressed genes in these pathways were related to the glioma (from 50% to 83%).

**Table 3:** The top 6 potential novel pathways (GeneGO) found from 4 datasets

Pathway Name	Object Count	Pubmed Count	Percentage(%)
Role of IAP-proteins in apoptosis	31	28	90.32%

Regulation of G1/S transition (part 1)	38	34	89.47%

NOTCH1-mediated pathway for NF-KB activity modulation	34	22	64.70%

Regulation of epithelial-to-mesenchymal transition (EMT)	64	54	84.38%

TGF-beta-dependent induction of EMT via SMADs	35	30	85.71%

Non-genomic (rapid) action of Androgen Receptor	40	27	67.50%

### Meta-analysis for pathway enrichment

Most meta-analysis methods developed currently for biomarker detection are just by combining genomic studies. By combining statistical significance at the gene level and at the pathway level, MAPE is a novel kind of meta-analysis approaches for pathway enrichment analysis [4]. In our work, MAPE has been applied to analyze the four gene expression datasets mentioned above to further verify our hypothesis. The pathway database of MAPE used in our study was GeneGO's MetaCore™, which could provide a better comparison with the results in our previous study [18]. In order to uncover the mechanism more accurately, we analyzed the data according to WHO grades. Accordingly, 91 pathways were found to be related to the glioma.

Combined the results obtained from the gene expression data, 27 common pathways were found both from microarray statistical analysis and meta-analysis. Moreover, the GeneGO's pathway for two results shows the same Ontology categories.

### Cross-validation by integrating other omics data

In order to verify our results, other two types of omics data were also integrated to analysis glioma. The discovery of microRNAs [19] introduced a new dimension in the understanding of how gene expression is regulated in 1993. MicroRNAs are a class of endogenous, single-stranded non-coding RNAs of 18-25 nucleotides in length, functioning as negative regulators of gene expression at the post-transcriptional level. The dysregulation of miRNAs has been demonstrated to play critical roles in tumorigenesis, either through inhibiting tumor suppressor genes or activating oncogenes inappropriately [20-22]. In particular, microRNA-21 (miR-21) has been reported to enhance the chemotherapeutic effect of taxol on human glioblastoma multiform cells [23]. For our purpose, three miRNAs expression profiles were downloaded from the GEO database, which are listed in Table 4. Owing to the different platforms of the datasets, the probe sequences were mapped to the miRBase (http://www.mirbase.org) by Blast [24] tools for identifying the concordant miRNA names. We again used the COPA package to detect the differentially expressed miRNAs between the normal and tumor samples. And the quantization of outlier extraction was set with the default parameters. The target genes for the significant miRNAs were predicted by four widely web-based databases, i.e. TargetScan, miRanda [25], RNAhybrid [26], and TargetSpy [27]. These tools were based on both miRNA sequences and 3'Untranslated Regions (UTRs) of protein-coding mRNA sequences and the binding energy calculated by the minimum free energy for hybridization. For deeper understanding target genes' biological functions, we mapped these targets of each dataset to GeneGO database for enriched biological pathways identification, respectively.

**Table 4:** Information on microRNA expression profiling data of glioma

Country	Platform	Number (all)	Sample information		MicroRNA Number	Publication Year
					
			Normal	Tumor		
Italy	DiSteBa_Homo sapiens_Glioblastoma miRNA 340_v1.0	74	37	37	340	2011.01

Italy	TJU-Human-Mouse-MicroRNA-1.6k-v1.1	35	13	22	353	2005.09

USA	Agilent 8 × 15K Human miRNA-specific microarray	34	10	24	1510	2009.12

According to three datasets of microRNAs data, 187 pathways were found to be associated with glioma when p-value < 0.05 was considered statistically significant. 5 out of the top 6 potential novel glioma pathways found in the gene expression profiles study also exit in microRNAs results, as listed in Table 5. Therefore, we suggest these 5 pathways would be putative novel glioma pathways. The GeneGO's Ontology categories of these pathways show the same result with that of gene expression datasets (Additional file 1).

**Table 5:** The top 5 novel GeneGO's pathways overlapped by gene and miRNA expression profiles

Pathway
Regulation of G1/S transition (part 1)

NOTCH1-mediated pathway for NF-KB activity modulation

Regulation of epithelial-to-mesenchymal transition (EMT)

TGF-beta-dependent induction of EMT via SMADs

Non-genomic (rapid) action of Androgen Receptor

ChIP-seq is another new technique for genome-wide profiling of protein-DNA interactions, histone modifications, or nucleosomes [28,29]. In ChIP-seq, the DNA fragments of interest are sequenced directly instead of being hybridized on an array. Compared with ChIP-chip, ChIP-seq offers significantly improved data with higher resolution, less noise, and greater coverage. Currently, this technology has been widely used to study transcription factor binding sites [30], and can provide invaluable information for studying gene regulation.

In our research, the ChIP-seq dataset (accession number GSM575227) from the study conducted by Fang [12] was downloaded as reads aligned to the human genome from the GEO database. Here, we detected significant peaks of signal enrichment with two different peak callers: MACS [31], SISSRs [32]. Default parameters were used in each case. The MACS uses a sliding window to scan the genome, and uses a locally estimated Poisson rate for enrichment peak identification. MACS not only found more peaks with fewer false positives, but also provided better binding resolution to facilitate downstream motif discovery. SISSRS is a novel algorithm for precise identification of binding sites from short reads generated from ChIP-seq experiments. SISSRs uses the direction and density of reads and the average DNA fragment length to identify binding sites. It detects points in the genome where the net difference between the forward and reverse read counts in a moving window transforms from positive to negative. It is more accurate, sensitive and robust for binding site identification compared with other approaches.

The overlapped significantly enriched peaks identified by the two approaches were used for subsequent analysis. We applied PeakAnalyzer [33] to assign the protein binding sites to target genes. Then the pathway analysis by mapping the genes to GeneGO got 76 glioma pathways with the 0.05 p-value. TGF-beta-dependent induction of EMT via SMADs, as one of the five pathways shown in Table 5, was surprisingly verified in the ChIP-seq analysis.

Lastly, we made a comparison among the pathways detected from gene expression data, MicroRNA expression data and ChIP-seq data, and the result show that 14 common pathways have been found in all the three different omics data (Additional file 2).

### TGF-beta-dependent induction of EMT via SMADs

**Figure 4:**
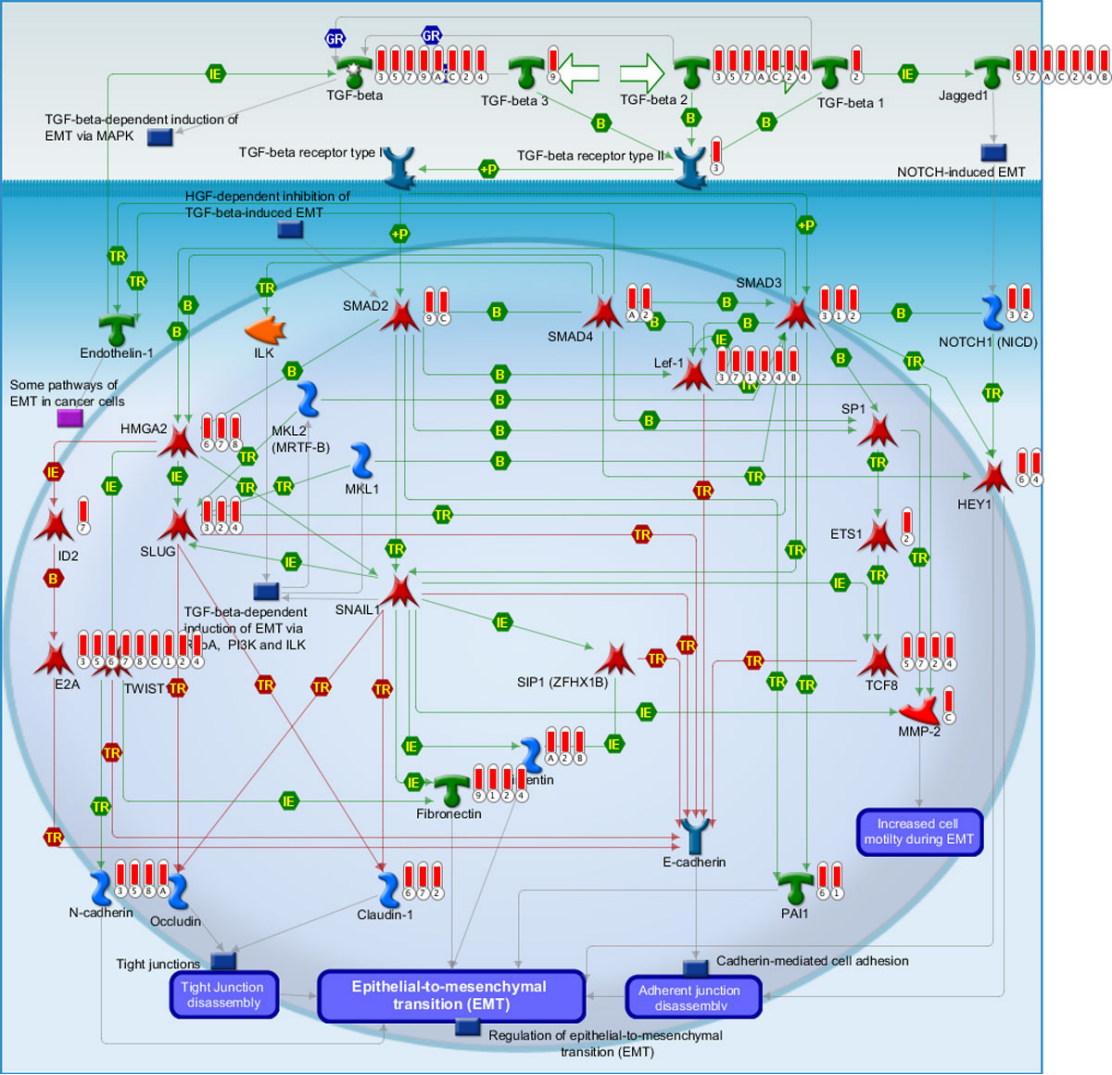
**GeneGo graphic illustration of TGF-beta-dependent induction of EMT via SMADs pathway**. The differentially expressed genes identified from the 12 groups of two-class are represented with red bar histograms. The numerical and alphabetic subscript represents the datasets to which the gene belongs.

**Figure 5:**
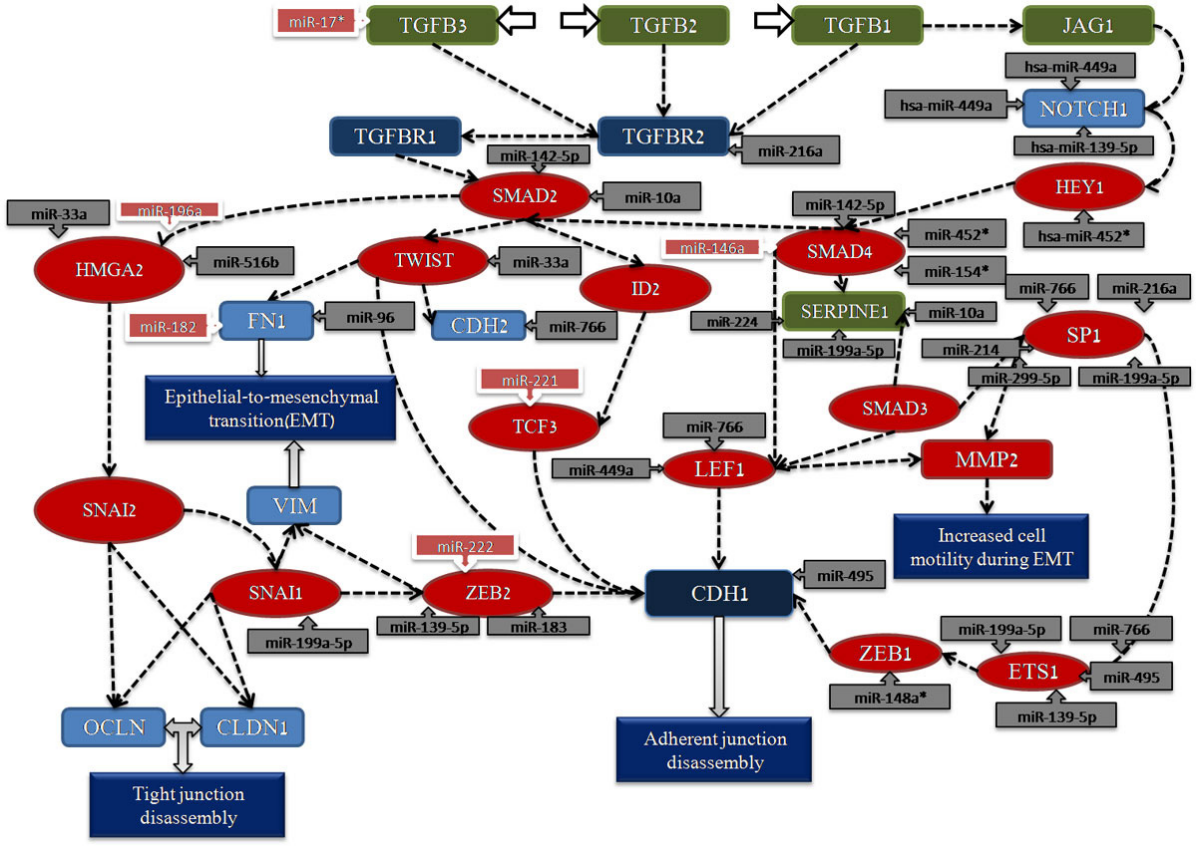
**A map for the key genes with microRNA information extracted from TGF-beta-dependent induction of EMT via SMADs pathway**. The differentially expressed genes identified from the pathway are represented by rectangle and oval, the red one denotes the TF, the dark blue one denotes receptors with enzyme activity, and the green one denotes receptor ligand. In addition, the rectangles with arrows means the microRNA that regulates the gene, and the ones reported as biomarkers are highlight with red color.

For the three types of "omic" data, one of the common pathways named TGF-beta dependent induction of EMT via SMADs was found. The pathway map for TGF-beta-dependent induction of EMT via SMADs in GeneGO is shown in Figure 4. Even in the same pathway, the differentially expressed genes may locate at different places, which supported our hypothesis again. Although such a pathway needs more biological experiments, it represents a good candidate for further study. The research result in the Entrez PubMed database showed that there is not any report about this pathway, so we check some identified important genes and build a pathway map that contains important microRNA information for the detail discussion. For example, Smad interacting protein 1 (SIP1) [34], TGF-beta [35], and LIF have been identified and play an essential role in glioma. Based on the systems biology level, we think the map with both gene and microRNA information from the differentially expressed analysis will produce more useful information. The pathway map, shown in Figure 5, includes the information of microRNAs that regulate genes. We hypothesize that microRNAs regulated some important genes in the pathway, which may served as biomarkers for glioma. Therefore, we searched these interesting microRNAs in the Entrez PubMed database, where some of them have been reported to be related with glioma. For example, Accumulating evidence indicates that miRNA expression can be used as a diagnostic and prognostic marker for human cancers. In Jiang's study [36], their results suggest that miR-182 could be a valuable marker of glioma progression and that high miR-182 expression is associated with poor overall survival in patients with malignant glioma. Zhang et al. [37] reported that miR-221/222 expression was significantly increased in high-grade gliomas compared with low-grade gliomas, and positively correlated with the degree of glioma infiltration. Therefore, the novel pathway, TGF-beta-dependent induction of EMT via SMADs, may play an important role to cause glioma occurrence.

## Discussion

Cancer is a type of complex disease [1], which means it caused by a combination of genetic perturbations, lifestyle effect and personal behaviours. Uncovering the molecular mechanisms of such complex disease, it requires a new paradigm that study cancer at a systems biology level, such as gene sets, dynamic network or pathway level. Till now, most of works just focus on the identification of individual genes which might play important roles in glioma carcinogenesis, such as YKL-40 is a biomarker in the series of GBM by the comparative expression patterns analysis [38]. In addition, CDK4, MDM2, EGFR, PDGFA, PDGFB and PDGFRA genes were suggested to be biomarkers for glioma, as well as CDKN2A, PTEN, RB1 and TP53 are found as the glioma suppressor genes. Despite of these known genes for glioma, the pathway analysis explore how genes interaction in a pathway to play their function. To this aim, we tried to find some new potential pathways based on the meta-analysed four gene expression profiling datasets on glioma.

Another additional difficulty of studying cancer relates of its heterogeneity at the molecular level. In heterogeneous disease, particular tumor, different cases will typically have different genes. Gene expression microarrays measure thousands of genes simultaneously; therefore, common statistical methods such as t-test will not work for finding these genes. The common significant gene analysis based on t-test or t-test like statistics methods have been used to study special genetic changes in glioma [39], and to identify some differentially expressed genes associated with glioma [40]. Fortunately, COPA, a novel method, has proven to be an effective approach to discover mechanisms underpinning heterogeneity in cancers by combined with pathway and functional analysis. We used COPA to identify the differentially expressed genes between glioma and normal samples in this study and then detected enriched gene sets and pathways via GESA tool and GeneGO's MetaCore™ software.

This pathway study was complemented with additional information including microRNA and ChIP-seq profiles. MicroRNAs analysis has rapidly become an attractive method for cancer research as it exhibits more accurate and sensitive compared with traditional gene expression profiling of mRNAs [41]. Accumulating evidence suggests some miRNAs play an important role in glioma occurrence. Han's study [11] demonstrated that β-catenin pathway regulates miR-21 expression via STAT3 playing a role in human glioma cell. Nowadays, with the decreasing cost of sequencing, ChIP-seq has become a useful tool for studying gene regulation and epigenetic mechanisms. ChIP-seq offers significantly improved data with higher resolution, less noise. Fang's work [12] demonstrated that SOX2 plays an important role in the carcinogenesis and development of glioma. And the target genes for SOX2 binding regions in glioma cells were identified, such as ARRDC4, PDE4D, BASP1 and so on. In our work, microRNA expression profiles and ChIP-seq data were integrated for the further verification. In comparison with the results from gene expression datasets, five novel glioma related pathways were also identified in these datasets. Within these pathways, some of them have already been reported as important pathways in glioma. By controlling transcription of the cyclin-dependent kinase inhibitor p27 (kip1), FOXO3a inhibits cell-cycle progression at the G1/S transition, which is frequently down-regulated in tumor cancers, such as human glioma. NF-kB is previously reported as a transcription factor, which controls expression of several oncogenes, growth factors and cell adhesion molecules and plays a key role in carcinogenesis [42-44]. Moreover, Li et al. [9] found that ECRG4 serves as a tumor suppressor in glioma in the NF-B pathway, which was supposed to be included in glioma cell growth suppression.

In conclusion, we proposed a novel meta-analysis based on systems biology level for cancer research and some putative novel pathways were found to be associated with glioma. Compared to previous analyses, our novel approach integrated three types of "omics" data including gene expression data, MicroRNA expression data and ChIP-seq data, which could perform cross-validation each other at the systems biology level, and thus the method is both possible and necessary to decrease the discrepancy and improve the understanding of the complex molecular mechanisms underlying cancer. The novel pathway, TGF-beta-dependent induction of EMT via SMADs, was found in all the profiling, and thus could serve as a candidate pathway for further experiment testing. We believed that the developed method and the identified new pathway in our work will provide more useful and detailed information for future studies at the system level.

## Conclusions

Systems biology provides powerful tools for the study of complex disease. System-based approach verified the idea that the overlapping of signatures is higher at the pathway or gene set level than that at the gene level. We have performed a pathway enrichment analysis by using GeneGo database, GSEA and MAPE software to show several novel glioma pathways. In addition, 5 out of these novel pathways have also been verified by integrating a wealth of miRNAs expression profiles and ChIP-seq data sets, thus, some good candidates for further study. This story would mark a beginning, not an end, to identify novel pathways of complex cancer based on systems level. Two valuable future directions would be rooted in the complexity and the heterogeneity of cancer. With the development of high-throughput technologies, more and more data should be considered and correlated at the level of systems biology. As was discussed in text, although many meta-analysis techniques and pathway enrichment analysis methods have been developed in the past few years, a more robust method by incorporating and evaluating these available methods is also needed immediately.

## Methods

### Dataset

We collected four publicly available glioma microarray expression datasets, which were performed using Affymetrix oligonucleotide microarray. All the datasets were generated by four independent laboratories. To obtain more consistent results, we proposed to meta-analyze the multiple microarrays. Rhodes et al. [45] indicated that multiple datasets should be meta-analyzed based on the same statistical hypothesis such as cancer versus normal tissue, high grade cancer versus low grade cancer, poor outcome cancer versus good outcome cancer, metastasis versus primary cancer, and subtype 1 versus subtype 2. Therefore, our meta-analysis on the basis of two types of samples, normal brain and glioma tissues, were comparable. The individual analysis of each dataset mainly includes three steps: pre-processing, differential expression analysis and pathway/gene set enrichment analysis. Most analysis processes were performed in R programming environment.

### Data pre-processing

The raw datasets measured with Affymetrix chips were analyzed using MAS5.0 [46] algorithm. We performed Median Absolute Deviation (MAD) method [47] for between-chip normalization of all datasets. Low-qualified genes were eliminated and the filter criterion was defined as 60% absence across all of the samples.

### Differential expression analysis

Cancer Outlier Profile Analysis (COPA) method was used for detecting differentially expressed genes between normal and tumor samples. The copa [11] package was implemented in R environments. Two steps of COPA statistic is defined as following: 1) the data was centered and scaled on a rowwise basis using median and median absolute deviation. The columns of microarray expression data matrix were samples and the rows were genes. 2) The data in the disease group was pre-filtered by setting the pre-filtration threshold as defaulted 95th percentile. It means that the genes with a number of outlier samples less than the 95th percentile were removed from further consideration. A threshold cut-off for 'outlier' status was set and applied to all genes.

### Pathway and gene set enrichment analysis

After COPA analysis, the interested genes were mapped to GeneGO database by MetaCore™ for pathway enrichment analysis. It is a most comprehensive and detailed human metabolism and signalling database [48]. In MetaCore™, the statistical significance (p-value) represents the probability to randomly obtain the intersection of certain size between two gene/protein datasets following hyper geometric distribution.

Additionally, we applied Gene Set Enrichment Analysis (GSEA) [14] to assess which gene set or pathway was significant. The method derives its power by focusing on gene sets, that is, groups of genes that share common biological function, chromosomal location, or regulation. GSEA used a collection of gene sets from the Molecular Signatures Database (MSigDB), which was divided into five major collections. In our work, we used C2 catalog of functional gene sets, which collected the signalling pathway information from the publicly available, manually curated databases and experimental studies.

Furthermore, we performed MAPE, a systematic approach improved by Shen [4] for pathway enrichment analysis. It provides a more robust and powerful tool by combining statistical significance across studies, and obtains more consistent results.

### Overlapping analysis at different levels

The overlapping analysis was performed between two-pair datasets on the same stage. For every pair of datasets, the number of significant genes, or pathways/gene sets was labelled as g1 in dataset-1, as g2 in dataset-2, respectively. The overlapping percentage between two datasets was designated as the number of overlapping genes/pathways divided by the number of genes, or pathways/gene sets in the union of g1 and g2. It can be calculated as follows:

$$\text{Overlapping percentage}=\frac{\text{s}}{\text{g1}+\text{g2 - s}}\times 100\%.$$

## Competing interests

The authors declare that they have no competing interests.

## Authors' contributions

YH carried out the computational analysis, YH, JL, WY, JC, GH and BS participated in the design and drafted the manuscript. BS and GH conceived and coordinated this study. All authors read and approved the final manuscript.

## Supplementary Material

Additional file 1 **GeneGO Ontology classification of 187 enriched pathways from the miRNAs expression profiles**. The Gantt bars described that these pathways could be divided into 26 GeneGO's Ontology categories. For example, 70 pathways were associated with Development, 16 pathways were relevant to Immune response, and 12 pathways were related to G-protein signalling and so on. Click here for file

Additional file 2 **The GeneGO's pathways overlapped by the omics data**. Click here for file

## Declarations

The publication of this article has been funded by the National Nature Science Foundation of China (31170795, 91230117, 91029703, 21203131), the Specialized Research Fund for the Doctoral Program of Higher Education of China (20113201110015). International S&T Cooperation Program of Suzhou (SH201120) and the National 973 Programs of China (2010CB945600).

This article has been published as part of *BMC Systems Biology *Volume 7 Supplement 2, 2013: Selected articles from The 6^th ^International Conference of Computational Biology. The full contents of the supplement are available online at http://www.biomedcentral.com/bmcsystbiol/supplements/7/S2.
