# Supplementary figures and images for: Identifying novel glioma associated pathways based on systems biology level meta-analysis
Source: BMC Syst Biol. 2013 Dec 17;7(Suppl 2):S9. doi: 10.1186/1752-0509-7-S2-S9 (PMC3866263; doi:10.1186/1752-0509-7-S2-S9)

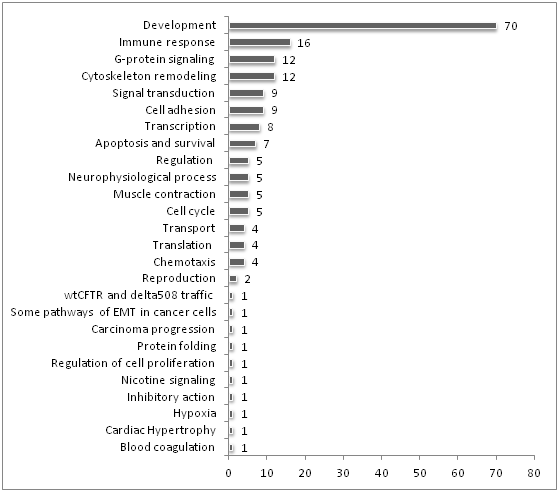

Supplement: Additional file 1 — GeneGO Ontology classification of 187 enriched pathways from the miRNAs expression profiles. The Gantt bars described that these pathways could be divided into 26 GeneGO's Ontology categories. For example, 70 pathways were associated with Development, 16 pathways were relevant to Immune response, and 12 pathways were related to G-protein signalling and so on. [file 1752-0509-7-S2-S9-S1.tif]
