# Supplementary material for: Identifying novel glioma associated pathways based on systems biology level meta-analysis
Source: BMC Syst Biol. 2013 Dec 17;7(Suppl 2):S9. doi: 10.1186/1752-0509-7-S2-S9 (PMC3866263; doi:10.1186/1752-0509-7-S2-S9)
Supplement: Additional file 2 — The GeneGO's pathways overlapped by the omics data. [file 1752-0509-7-S2-S9-S2.docx]

| **NO.** | **GeneGO pathway name** |
| --- | --- |
| 1 | Beta-2 adrenergic receptor anti-apoptotic action |
| 2 | cAMP signalling |
| 3 | Chemokines and adhesion |
| 4 | CXCR4 signalling pathway |
| 5 | EGFR signalling via small GTPases |
| 6 | GPCRs in the regulation of smooth muscle tone |
| 7 | Inhibitory action of Lipoxin A4 on PDGF, EGF and LTD4 signalling |
| 8 | Leukocyte chemotaxis |
| 9 | Lipoxin inhibitory action on PDGF, EGF and LTD4 signalling |
| 10 | Regulation of CFTR activity (norm and CF) |
| 11 | Relaxin signalling pathway |
| 12 | Reverse signalling by ephrin B |
| 13 | S1P1 signalling pathway |
| 14 | S1P2 receptor signalling |
